# Reliable Location-Based Services from Radio Navigation Systems

**DOI:** 10.3390/s101211369

**Published:** 2010-12-13

**Authors:** Di Qiu, Dan Boneh, Sherman Lo, Per Enge

**Affiliations:** 1 Aeronautics & Astronautics Department, Stanford University, Durand Building, Stanford, CA 94305, USA; E-Mails: daedalus@stanford.edu (S.L.); penge@stanford.edu (P.E.); 2 Computer Science Department, Stanford University, Gates 475, Stanford, CA 94305, USA; E-Mail: dabo@cs.stanford.edu (D.B.)

**Keywords:** Loran-C, location-based security, location tag

## Abstract

Loran is a radio-based navigation system originally designed for naval applications. We show that Loran-C’s high-power and high repeatable accuracy are fantastic for security applications. First, we show how to derive a precise location tag—with a sensitivity of about 20 meters—that is difficult to project to an exact location. A device can use our location tag to block or allow certain actions, without knowing its precise location. To ensure that our tag is reproducible we make use of fuzzy extractors, a mechanism originally designed for biometric authentication. We build a fuzzy extractor specifically designed for radio-type errors and give experimental evidence to show its effectiveness. Second, we show that our location tag is difficult to predict from a distance. For example, an observer cannot predict the location tag inside a guarded data center from a few hundreds of meters away. As an application, consider a location-aware disk drive that will only work inside the data center. An attacker who steals the device and is capable of spoofing Loran-C signals, still cannot make the device work since he does not know what location tag to spoof. We provide experimental data supporting our unpredictability claim.

## 1. Introduction

When a device wishes to determine its position, it does two things. First, the hardware uses an antenna and receiver to capture and record a location measurement. Second, the location measurement is converted into a global position in the form of longitude and latitude. Most often these two steps are conflated, and both are seen as necessary to enable location-based applications. In this paper we argue that for many security applications only the first step is needed: there is no need to accurately map the location measurement to an accurate global position. Therefore, these location-based security applications can be implemented using a variety of radio frequency (RF) signals, including broadcast communication signals, such as AM/FM, cellular, DTV, Wi-Fi, *etc.*, navigation signals, and an integration of various signals.

While GPS provides accurate position data, other location services are far less accurate. LOng RAnge Navigation (Loran) [1], for example, uses a 3 km wavelength, and standalone Loran has an absolute accuracy of several hundred meters. Loran-C, the most recent version of Loran in use, is a terrestrial navigation system originally designed for naval applications and its operation is described in Appendix A. Its modernized version, called enhanced Loran (eLoran) [2], together with differential corrections can achieve an accuracy of 8 to 20 meters. This paper uses standalone Loran-C, which has good repeatable accuracy, but low absolute accuracy, as a case study and shows that high absolute accuracy is not a requirement for a number of location-based security applications. As with all radio-based systems, Loran-C radio signals are distorted by buildings and other objects, causing measurements to change greatly over short distances. Our main result shows that one can exploit these chaotic changes to obtain a precise and reproducible location tag with an accuracy of about 20 meters. Reproducibility means that measurements at the same location at different times always produce the same tag. While there is no way to map location measurements to an accurate position, there are still many applications, primarily security applications, for which a reproducible and precise tag is sufficient.

We build a reproducible and precise tag using recent results from biometric authentication for location-based security applications. In particular, we rely on fuzzy extractors [3,4] and secure sketches [5,6], originally designed for fingerprint-based authentication. The idea is to store some public information that enables anyone to convert an erroneous measurement into a consistent tag. We develop specific fuzzy extractors designed to handle radio-type errors. The challenge is to correct for signal variations due to day/night, humidity, and seasonal changes.

Although the absolute accuracy of standalone Loran-C is not comparable to that of GPS, it has several advantages over GPS for security applications. First, Loran-C is a high power terrestrial signal and easily penetrates buildings and cities. GPS, which has extremely weak signals below the thermal noise floor, is usually not available inside buildings. Second, we show that our radio-based location tag is unpredictable at a distance: an attacker who is several hundred meters away from a target location cannot predict the tag at the target. The reason is that signal attenuation by buildings is chaotic and difficult to interpolate. We give experimental evidence for this claim and discuss some applications. In contrast, GPS is, by design, predictable from a great distance and consequently GPS cannot be used for applications that require a secret location tag. Third, Loran-C has good regional coverage in Northern Europe and much of East Asia, like China, Japan, and Korea. Although the transmission of Loran-C signals in North America has been terminated in February 2010, the decision with eLoran has yet to be made. eLoran will have a data channel [2]. While some uses of the data have been defined, others have not. Therefore, several message types have been left unassigned to support useful application such as location-based security in the course of eLoran design. Loran antenna size may have been a practical issue in many applications. Recent research has shown that a miniature H-field antenna of 2 × 2 cm can be achieved [7]. With this size, a Loran H-field antenna can be easily fit into a number of portable electronic devices.

Our goal is to develop a standardized process to quantify the precision, reproducibility and security of a location tag for security applications. The design and implementation of fuzzy extractors for location-based security discussed in this paper will apply to all radio-based signals. We use Loran-C as a convenient example and evaluate the location tag performance using real data.

### 1.1. Security Applications

We discuss a number of security applications where the properties of our location tag—precise, reproducible, and unpredictable at a distance—come into play. We reiterate that although our tag is very sensitive to spatial changes, it cannot be used to obtain position information (latitude/longitude) of that accuracy. In all our examples we assume the device is tamper resistant so that bypassing the location tag check is difficult.

#### 1.1.1. Digital Manners Policies (DMP)

Technologies for digital manners (DMP) [8] attempt to enforce manners at public locations. A DMP-enabled cell phone can be programmed by the phone provider to turn off the camera while inside a hospital, a locker room, or a classified installation. Or the phone can be programmed to switch to vibrate mode while inside a movie theater. Many other applications have been considered. Although these ideas are highly controversial [9], we only focus on the technical contents and feasible implementation of the ideas.

To implement DMP one assumes that the device needs to know its precise location. We argue that this is incorrect. Using our radio-based tag, one can build a list of location tags where the camera is to be turned off. The device downloads an updated list periodically. When the device encounters a location tag on this blocklist, it turns the camera off. When the device leaves the blocked location the camera is turned back on. Hence, digital manners are enforced without ever telling the device its precise location.

A DMP system must survive the following attack: the attacker owns the device and tries to make the device think it is somewhere else. Since most places are not blocked, any location confusion will do. To survive this threat any location-based DMP system must make the following two assumptions:

- First the device, including the antenna connection, must be tamper resistant. If the antenna connection is not protected then anyone can tamper with signals from the antenna. The simplest attack is to add a delay loop to the antenna. Since location measurements are time based, the delay loop will fool the device into thinking it is somewhere else.
- Second, it should be difficult to spoof the Loran-C radio signals by transmitting fake signals from a nearby transmitter. The safest defense against spoofing is cryptographic authentication for Loran-C signals. Qiu *et al.* [10] proposed a clever method for embedding TESLA [11] authenticators into Loran-C signals to prevent spoofing. We point out that even without cryptography, spoofing Loran-C signals is far harder than spoofing GPS: In fact, GPS spoofers are commercially available and are regularly used by GPS vendors for testing their products.

Both assumptions are necessary to build an effective DMP system regardless of the navigation system used. Our goal is not to promote DMP but rather to show that an accurate DMP system can be built from standalone Loran-C signals.

#### 1.1.2. Location Based access Control

While DMP is a blocklisting application, access control is a whitelisting example. Consider a location-aware disk drive. The drive can be programmed to work only while safely in the data center. An attacker who steals the device will not be able to interact with it.

We consider two attack models:

- *Private locations:* suppose the device is located in a guarded data center and the attacker has no access to the insides of the data center. The attacker steals the device (say, while in transit [12]) and tries to make the device think it is still in the data center.
- *Public locations:* in this case the attacker has complete access to the data center and the attacker can measure the authorized location tag. After stealing the device the attacker can try to spoof the Loran-C signal to make the device think it is still in the data center. Unlike the DMP application where any location confusion was sufficient for the attacker, here the attacker must cause the device to think it is precisely in the right place in the data center, with 20 meter accuracy. Simply adding delay loops to the antenna will not work.

In both threat models we must assume that the device is tamper resistant. Otherwise, the attacker can simply modify the device and bypass the location check. In the case of a public location we must also assume cryptographic authentication on Loran-C signals, as discussed in the DMP application.

Interestingly, for the private location settings, the unpredictability of the Loran-C location tag implies that we do not need any signal authentication nor do we need to protect the antenna connection to the device. In Section 4 we show that if the attacker has never been in the data center then he cannot tell for sure what location tag should be supplied. We show that even if the attacker takes many measurements several hundreds of meters away (say in the parking lot) he still cannot tell for sure what tag to supply.

One option available to the attacker is to build a list of candidate location tags and try them one by one. In Section 4 we show that the list would need to include several dozen candidate tags. But the device can easily shutdown if it ever receives a sequence of incorrect location tags. Consequently, a trial and error attack will not get very far.

We note that location-based access control using encryption was studied by Scott and Denning [13] under the name Geoencryption, which uses physical locations, such as latitude, longitude and altitude measurements from GPS, for security applications. Our location tag derived from raw location measurements is more unpredictable and provides more information entropy,

## 2. Background on Fuzzy Extractors

In the previous section we showed applications for a precise and reproducible location tag. We now show how to build such tags using standalone Loran-C system. To ensure that our tags are reproducible we will make use of fuzzy extractors [3,5]. Fuzzy extractors were originally designed for biometric authentication systems. Since biometric scanners introduce errors, one needs same way to extract a reproducible tag from the scanner’s output. While biometric fuzzy extractors are designed with a specific error model in mind, here we need a fuzzy extractor tailored for the Loran error model.

### 2.1. Fuzzy Extractors: Definitions

We follow the definitions in [5]. Measurements live in a set *M* which is equipped with a distance function denoted dis. Roughly speaking, dis(*x,y*) is small if *x* is “close” to *y*.

**Figure 1.**
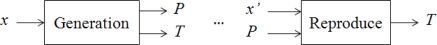
Fuzzy extractor in action.

*Fuzzy extractor*. A fuzzy extractor works in two steps, as shown in Figure 1. During the registration step one runs algorithm Gen on input *x*∈M to generate a public value *P* and a tag *T*. Later, given a noisy version of *x*, denoted *x’*, one runs algorithm Rep on input *x’* and *P* to reproduce the tag *T*. The idea is that if *x* and *x’* are fingerprint scans of the same finger, then *x* is “close” to *x’* and both should produce the same tag *T*. If *T* has sufficient entropy then it can used as a login password. Clearly we require that *P* reveal little or no information about the tag *T*.

*Definition 1.* A fuzzy extractor is a tuple (*M*, *t_0_, t_1_*, Gen, Rep), where *M* is the metric space with a distance function dis, Gen is a generate procedure and Rep is a reproduce procedure, which has the following properties:

If Gen(*x*) outputs (*T*, *P*), then Rep(*x’*, *P*) = *T*, whenever dis(*x*, *x*’) ≤ *t_0_*. If dis(*x*, *x*’) ≥ *t_0_*, then there is no guarantee *T* will be output. In addition, if dis(*x*, *x*’) ≥ *t_1_*, Rep(*x*’, *P*) = *T’*, and *T’ ≠ T*.

### 2.2. Known Constructions for Fuzzy Extractors

Initial constructions were proposed by Juels and Wattenberg [3]. Their scheme uses an error correcting code to handle the hamming metric on binary data. Juels and Sudan [4] provide a fuzzy extractor for the set difference metric, which is the first construction for a non-hamming metric. Dodis *et al.* [5] give precise definitions for the problem and provide constructions for hamming distance, set distance and edit distance.

All these schemes primarily apply to binary data which does not fit our settings where location measurements are vectors of real numbers. One exception is a construction of Chang and Li [14] that can be adapted to give a fuzzy extractor for the scenario where one of the Loran-C transmitters is offline (e.g., for maintenance).

## 3. Generating a Reproducible and Precise Location Tag from Loran-C

Our goal is to build a reproducible and precise tag from standalone Loran-C measurements. We first explain what a Loran-C measurement looks like and then discuss the error model for these measurements. Finally, we present a simple fuzzy extractor for this error model.

*Loran-C measurements.* Radio-based navigation uses signals from multiple transmitters to estimate the receiver’s positions. Loran-C is a terrestrial, low frequency pulsed navigation system that operates in much of the northern hemisphere and uses static transmitters. Four transmitters on the west coast of the US, called the west coast Loran chain (GRI9940) are used for navigation in the western US. These four stations are located at Fallon, NV; George, WA; Middletown, CA; and Searchlight, NV. Pulses from this chain are broadcast every 0.0994 seconds. Fallon is the master station and the remaining three follow in sync. From each station we obtain three values, called *features*, per pulse:

- Time-of-arrival (TOA) or time difference (TD): measures the propagation time from the transmitter to the receiver,
- envelope-to-cycle difference (ECD): measures carrier propagation rate, and
- signal-to-noise ratio (SNR).

An example measurement from the Middletown, CA station taken at Stanford is a triple:

(496.8 microseconds, −0.145 microseconds, 41 dB)

The exact meaning of these numbers is not important for our discussion here. What is important is that each transmitter produces a triple of real numbers (features) per pulse. Collecting the signals from all four stations gives a 12-dimensional real vector from which we wish to derive a location tag.

**Figure 2.**
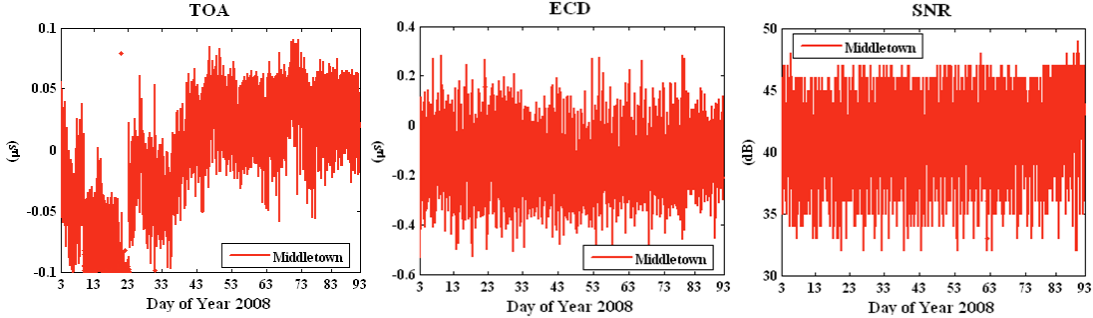
Stanford seasonal monitor data for 90-day period for Middletown, CA: **(a)** TOA; **(b)** ECD; **(c)** SNR.

*Loran-C error patterns.* Due to measurement errors and environmental changes, taking multiple measurements at the same location, but at different times, produces different 12 dimensional vectors. Figure 2 shows temporal variations in the triple (TOA, ECD and SNR) as measured from the Middletown station over a 90 day period. These measurements were taken at Stanford, CA. The wild swings in TOA, for example, reflect seasonal variations between winter and spring. We next explain the reason for these variations and how to model them.

- The most common error source is the thermal noise in all electronic devices, considered as white Gaussian noise. This noise cannot be eliminated and is always presenting in all electronic devices and transmission media.
- Many environmental factors cause signal variation, including temperature changes between night and day, changes in soil conductivity over time, humidity, local weather, *etc.* [15]. In particular, temperature and humidity variations have a considerable effect on propagation speed. The extra delay in propagation time or TOA can introduce a position error of hundreds of meters [16]. This particular error source in Loran is called additional secondary factor (ASF) and represents one of the largest error sources in Loran.
- Location vectors are continuous and need to be quantized. Quantization error, which is the difference between value of continuous feature and the quantized value, can lead to errors in the derived location tag. The quantization error is usually correlated with the two types of errors discussed above.
- The last type error results from maintenance of any radio-based system. A transmitter can go offline, in which case we lose all measurements associated with that station. Ideally, we would like this to have no effect on the location tag produced by our system.

A fuzzy extractor for Loran signals must take seasonal variations into account and can correct errors differently depending on the time of year.

## 3.1. Fuzzy Extractors for Location-Based Services

### 

#### Construction 1: Fuzzy extractor for Euclidean distance

We propose a fuzzy extractor when all Loran-C transmitters are present. Thus the features are real numbers over *R* and Euclidean distance is sufficient for the distance metric. Let *x* be a location feature vector at registration while *x’* be the feature vector at verification time, Δ is the step size to quantize the feature. The distance dis(*x*, *x*’) can be bounded by adequate threshold. This threshold, δ, can be a design parameter. We need to develop a fuzzy extractor that can reproduce tag *T* when the errors |*x − x’*| ≤ δ. This fuzzy extractor is designed to tolerate the random noise, biases and quantization errors.

Let the metric space *M* = [*A*_i_, *B*_i_]^n^, *n* = 12 if we use the triple from four Loran-C stations. Thus *x*, *x’* and Δ are vectors that have *n* dimensions. The quantization step Δ is a design parameter and chosen by a user. We will discuss how to choose reliable Δ in Section 3.2. We consider the distance measure for Loran-C features is L_∞_ norm to be conservative:

$$\text{dis}(x,\;x^{\prime })={\left(\underset{i}{\text{max}}\frac{\left|x_{i}-x_{i}^{\prime }\right|}{\Delta_{i}}\right)}_{i=1}^{n}$$

The construction of fuzzy extractor for Euclidean distance is as follows: during registration, feature vector *x* is quantized to get *T* and store public value *P*, whereas, during verification, given a slightly different location feature *x’* and *P*, compute *T’. P*, *T* and *T’* are also n-dimensional vectors. *P*_i_ represents the *i*_th_ feature in vector *P*. The elements in vector *T* are integers but they are not necessarily positive. For instance, it is possible to result in a negative TD if the distance between the secondary station and a user is shorter than the distance between master station and the user. The basic idea of this fuzzy extractor is to adjust the offsets between the continuous features and the discrete ones due to quantization:

- $\text{Gen}(x)=\left(\begin{array}{c} T={\left\lfloor \frac{x_{i}}{\Delta_{i}}\right\rfloor }_{i=1}^{n}\\ P={\left(x_{i}-\Delta_{i}\left\lfloor \frac{x_{i}}{\Delta_{i}}\right\rfloor \right)}_{i=1}^{n} \end{array}\right)$
- $\text{Rep}(x^{\prime },\;P)={\left\lfloor \frac{x_{i}^{\prime }-P_{i}+\frac{\Delta_{i}}{2}}{\Delta_{i}}\right\rfloor }_{i=1}^{n}=T^{\prime }$

*Claim 1.* If $\text{dis}(x,\;x^{\prime })<\frac{1}{2}$, then tag *T* can be reproduced, that is, *T’* = *T*. This claim defines the reproducibility of location tags. If *x’* is measured at the same location of *x*, we can reproduce *T* when the distance of *x* and *x’* is less than Δ/2.

*Claim 2.* If *dis*(*x*, *x*’) ≥ *t*_1_, then tag *T’* ≠ *T*. This claim defines the precision of locations tags. If *x’* is measured at a different location but close to the location of *x*, it is not expected that *x’* achieves the same tag as *x*.

It is easy to see that our construction is a fuzzy extractor (as in *Definition 1*).

#### Construction 2: Secret Sharing Based Fuzzy Extractor for Hamming Distance

The distance metric in this construction is hamming. The input to the fuzzy extractor is quantized feature vector *q_x_* instead of *x*, where $q_{x}={\left\lfloor \frac{x_{i}}{\Delta_{i}}\right\rfloor }_{i=1}^{n}$ is *n*-dimensional. The scheme is based on the property of secret sharing: a secret can be reconstructed given a subset of shared information. The construction is as follows:

- Create a polynomial *f*(*x*), such that *f*(*i*) = *q_xi_*, ∀ *i* = 1, 2, …, *n.*
- Let *m* be an integer and *m* < *n*.
- $\text{Gen}(x)=\left(\begin{array}{c} T=<f(1),\;f(2),\ldots ,\;f(m)>\\ P=<f(j),\ldots ,\;f(j+n-m-1)> \end{array}\right)$ where, *j*,…,*j*+*n*–*m*+ 1 ∉ {1,…,*n*}
- $\text{Rep}(x^{\prime },\;P)=\left(\begin{array}{c} f^{\prime }(x)\\ T^{\prime }=<f^{\prime }(1),\;f^{\prime }(2),\ldots ,\;f^{\prime }(m)> \end{array}\right)$

*Claim 3.* If *dis*(*q_x_*, *q_x_*’) ≤ n − m, then tag *T* can be reproduced. When the hamming distance between two vectors is less than *n − m*, the polynomial *f*(*x*) can be reconstructed with the assistance of *P* thus *T’* = *T*.

*Claim 4.* If *dis*(*q_x_*, *q_x_*’) > n − m, then tag *T’* ≠ *T*. The precision of location tag *T* relies on the features *x_1_*,..,*x_m_*. This construction increases reproducibility but reduces entropy because we only use *m* out of *n* features to compute a tag.

### 3.2. Experimental Results

In this section we use real standalone Loran-C data to evaluate the precision and reproducibility of Loran-C location tag and evaluate the effect of the Euclidean metric fuzzy extractor. We performed two experiments: (1) data was collected at various test locations to examine the precision of tags, and (2) data was collected at one location over 90-day period to study the reproducibility of tags.

#### Data at Different Locations Evaluating Tag Precision

**Figure 3.**
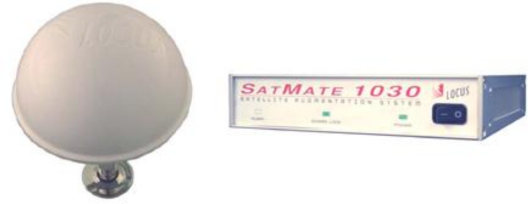
Loran-C H-field antenna (left); SatMate receiver (right).

We selected three different environments, where our proposed location-based security applications may occur, to perform the precision test: parking structure, soccer field and office building. At each location we used multiple test points for five minutes at each test point. An H-field antenna and Locus Satmate receiver, shown in Figure 3, were used for the data collection. The receiver averages and outputs Loran location features every minute.

- *Scenario 1.* The first data set was collected at 21 different test points on the top floor of a parking structure at Stanford University. This place has open sky view and no obstruction from the environments but there are some metal structures nearby. The altitude is relatively high compared with the other two scenarios. The dimension of the parking structure is approximately 70 × 50 meters.
- *Scenario 2.* The second data set selected 16 test points in a soccer field. This environment has some obstructions from trees and buildings. The field has a dimension of 176 × 70 meters so the distribution of the test locations are less dense compared to the other two scenarios.
- *Scenario 3.* The third data set, which includes 21 test points, was collected on the top floor both inside and outside a building. The concrete building with metal frames attenuates signal strength more but introduces more uniqueness in the location features, which can be beneficial to the computation of location tags.

We used the triple (TD, ECD, SNR) from four stations in the west coast chain (GRI 9940). Quantization steps are chosen based on the measured SNR. Low SNR signals are often attenuated more and pick up more noise. In general, features from low SNR stations are less consistent; thus larger quantization steps should be applied. We then created two-dimensional cells using Voronoi diagrams and mapped the tags into the cells accordingly. The color map is superimposed on the Google map. A color bar is used to label the hexadecimals of the first 16-bit of tag. This distribution plot can help us visualize how location tag varies in a two-dimensional view. Each black dot together with the numbered label at the center of the cells represents a test location.

**Figure 4.**
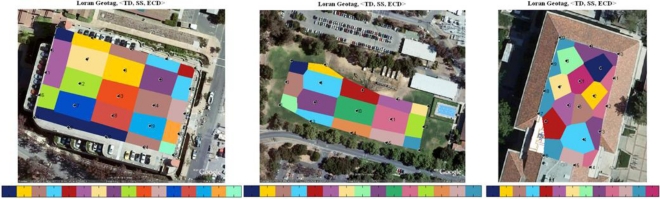
Visualization of location tags: **(a)** parking structure (left); **(b)** soccer field (middle); **(c)** Durand building (right).

The left of Figure 4 is the tag plot on the top floor of the parking structure, the middle plot represents the results of a soccer field, and the right plot shows the top floor/roof of Durand building. Loran signals are very sensitive to the environment, especially to metal structures. The re-radiation of signals from metals can cause more distortion to the RF signals thus higher precision or spatial variation of tags at certain locations. We observe this from the location tag maps of scenario 1 and scenario 3. The locations with very small separations still result in different location tags. It is worth to mention that only two stations, Fallon and Middletown, are used to compute tags for scenario 3, while the other two scenarios use all four stations from GRI 9940. Due to the low signal strength indoors, the SatMate receiver was not able to acquire the other two low SNR stations, George and Searchlight. The averaged precision of three different scenarios is as follows:

- The precision of Loran-C tags in the parking structure ranges from 8 meters to 35 meters. There are four locations that resulted in the same tag shown in dark blue on the left of Figure 4.
- The precision of tags in the soccer field is lower compared with that of the parking structure due to the large separations between the selected test locations or insufficient number of test points used. The averaged size of the colored cells that represents location tag is approximately 30 × 50 meters.
- Although the indoor signals are not good enough to solve a position fix because low-SNR signals are not able to track. The generation of a location tag does not rely on the solved position fix as the location tags are derived from location-dependent features. As a result, it is not required to have more than four transmitters to implement location-based security although more transmitters would provide more information entropy or longer tag to the system. The smallest colored cell or the highest tag precision in this indoor scenario is approximately 5 meters depicted in purple in the middle of the right plot in Figure 4. An upper bound on actual tag precision at this location is the largest cell, 8 × 20 meters.

#### Data at One Location Evaluating Reproducibility

In this section we use the seasonal data shown in Figure 2 to compare the reproducibility of a location tag with and without a fuzzy extractor. Again same triple is used in this experiment. We use TD instead of TOA to minimize the impact of ASF errors: TOA of the master station is used as a reference to mitigate the temporal variations of secondary stations. Our experiments show that the standard deviation of TOA from Middletown is 12.19 meters and the standard deviation of TD from Middletown is reduced to 3.83 meters [17]. However, TD provides less information entropy in comparison with TOA as we lose the TOA entropy from master station.

**Figure 5.**
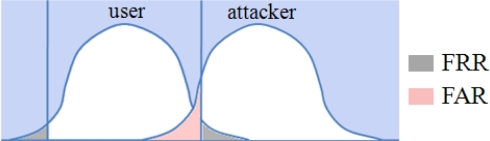
Performance metrics illustration.

*Performance metrics*. Before we discuss the experimental results from the seasonal data we introduce the performance metrics that help to quantify and measure the reproducibility of a location tag. The problem of deciding whether the derived location tag is authentic or not, can be seen as a hypothesis testing problem. The task is to decide which of the two hypotheses H_0_ (accepting as an authorized user) or H_1_ (rejecting as an attacker) is true for the observed location measurements. Location-based system makes two types of errors: (1) mistaking the measurements or derived tag from the same location to be from two different locations and accepting hypothesis H_1_ when H_0_ is true, called false reject; and (2) mistaking the measurements or derived tags from two different locations to be from the same location and accepting H_0_ when H_1_ is true, called false accept. Both false reject rate (FRR) and false accept rate (FAR) depend on the accuracy of equipments used, step sizes chosen to quantize location features and environmental conditions. These two types of errors can be traded off against each other by varying the quantization steps. A more secure system aims for low FARs at the expense of high FRRs, while a more convenient system aims for low FRRs at the expense of high FARs. Figure 5 illustrates the two error rates of location tags with the assumption that the probability distributions are Gaussian, which is not necessarily true in practice. The grey tails represent the false reject of an authorized user while the red area is the false accept of an attacker.

**Figure 6.**
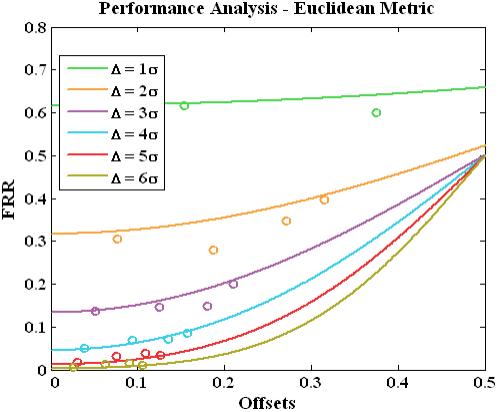
FRR of a location feature.

*Choosing a reliable quantization step for a location feature.* Users’ false reject rate significantly depends on the standard deviation of the features. Large standard deviation implies high temporal variations; thus the distance between the received features at verification and the ones at registration might be large. Therefore, the quantization step should be chosen to be proportional to the standard deviation σ of features. In this analysis we show that the quantization step has to be larger than 4σ to achieve reasonably small FRR, less than 0.1. The FRR analysis is illustrated in Figure 6. The quantization step ranges from σ to 6σ. The x-axis is the feature offset between registration and verification. The y-axis is the estimated FRR. The solid lines are analytical results and we assumed the distribution of location feature is near-Gaussian after the ASF mitigation. The dots are derived using the seasonal data. We used ECD from four stations in this experiment. To estimate FRR we take the first day of the 90-day ECD data as registration to compute a location tag and the data from the rest of 89 days for verification. The experimental FRR is the number of days, in which the tags are matched with the registered tag on day one, divided by 89. The experimental results match well with the analytical curves. As expected, FRR increases as offset goes up and quantization step goes down.

*Using multiple features.* The derived FRR in Figure 6 only represents the error rate of one particular location feature. Practically, multiple features are used to achieve more entropy, precision and higher difficulty in predicting the desired tag. However, one drawback using multiple features is that the FRR of the system is increased or reproducibility is reduced. The system FRR can be estimated as $\prod_{i=1}^{n}p_{i}$ if we assume the location features are independent from each other, where *p_i_* is the error rate of one feature. Practically, location features are slightly correlated in some environments. For instance, the signal strength is inversely proportional to the propagation distance, which is determined by TOA. This is true when the antenna is placed in an open sky area and has no obstructions from surroundings. To solve the reliability problem using multiple features, secret sharing based fuzzy extractor can be used together with the Euclidean metric fuzzy extractor. Only a subset of features is used to compute tags thus the total FRR is limited.

**Figure 7.**
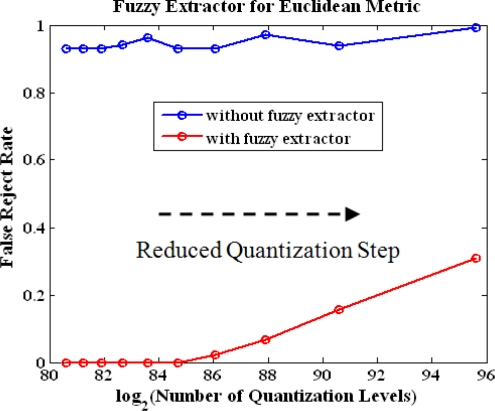
Performance of Euclidean metric fuzzy extractor.

*Euclidean metric fuzzy extractor performance of multiple features.* Now we use the triple from four stations to evaluate experimentally the performance of Euclidean metric fuzzy extractor. We reduce the quantization steps of the features gradually to observe the change of FRR and the number of quantization levels, which determine the entropy of location tag. The plot is shown in Figure 7. The blue line represents the FRR without the use of the fuzzy extractor while the red line is the results using the fuzzy extractor. As expected, the FRR is dramatically reduced after the use of the fuzzy extractor. The fuzzy extractor guarantees the measurements lying in the center of quantization interval. The graph shows that we can achieve total entropy of 86 bits with FRR is less 0.1 with adequate quantization steps.

## 4. Loran-C Tags are Unpredictable

Next we ask whether Loran-C tags are predictable from a distance. In this paper unpredictability refers to the difficulty of an individual in predicting the Loran measurements at a given time and place. The temporal variations due to propagation path delay variations and skywave as well as the unexpected distortions in the RF signals due to local features such as buildings and large metallic structures can introduce randomness and entropy in the generation of a location tag, which makes attackers to take more time and effort to break into the system.

We discussed applications for this unpredictability test in Section 1.1. To justify the claim that Loran-C tags are unpredictable, we perform two experiments. While we cannot prove the difficulty of prediction mathematically as it is not possible to come up a universal model that suits for all the environments; however, we can show the nonlinear of the Loran-C features experimentally. The predictions can be based on path propagation, reflection, diffraction, diffuse wall scattering and transmission through various materials. The sum of all the components is taken to get TD, ECD and SNR. Moving objects like people can cause not only attenuation but also fluctuation. The irregularities make the prediction even harder.

**Figure 8.**
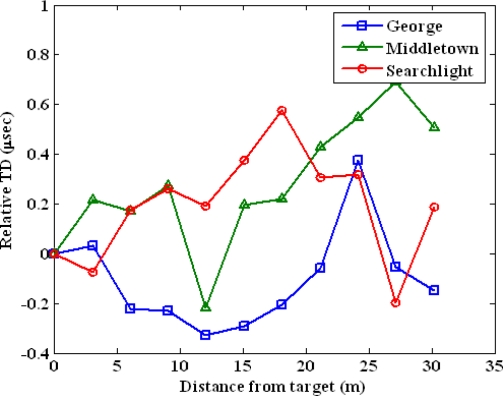
Spatial variation of TD measurements collected in a parking structure.

We perform the following two experiments to test the difficulty to predict a location tag. The first experiment uses the data set collected in a parking structure from 11 test points. The test locations are lined up in one dimension and the separation between adjacent points is approximately three meters. We chose the first point as our target or user location. Figure 8 plots the spatial variations of TD of George, Middletown and Searchlight. The x-axis is the measured distance of test points from the target point. The y-axis is the relative TD in microseconds. We zeroed out the means of the TDs to achieve the same scale for the measurements from three stations. The nonlinearity of the Loran-C measurements is clear from the graph. Low-SNR stations, George and Searchlight, are attenuated more from the obstructions in the environment compared to the strongest station Middletown. This results in more nonlinear variations in the low-SNR stations.

**Figure 9.**
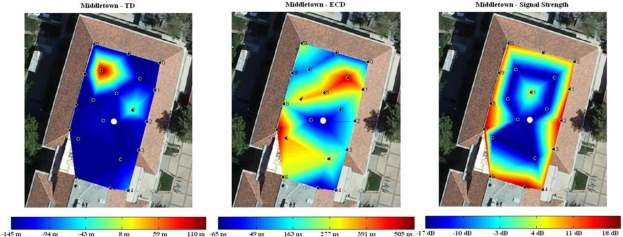
Spatial variation of location data from Middletown in Durand building: **(a)** TD; **(b)** ECD; **(c)** Signal strength.

The second experiment uses the same data set collected in Durand building for the precision test discussed in Section 3. We chose the center point as our target point and measured Loran-C features with increasing distances from the target point. The point is shown as white dots in the plots of Figure 9. The color contour plot is again superimposed on the Google map. The color bar shown at the bottom represents feature values of various locations. Figure 9 illustrates the spatial variations of TD, ECD and Signal strength measured from Middletown. If feature variations are linearly proportional to distance, the color of the map should change from blue to red gradually with equal diameter. We observe that ECD are more nonlinear in comparison with TD and signal strength because phase is very sensitive to building structures and environments. The non-linearity of location features can significantly benefit the design of location-based security applications as it results in the features are highly unpredictable.

## 5. Conclusions

We showed that a radio navigation system with high absolute accuracy and low repeatable accuracy such as standalone Loran-C can be used to generate a precise and reproducible location tag. A location tag is computed from location-dependent features and can be used for a number of security applications. A location tag is not a replacement but builds on the conventional security schemes. We discussed applications to DMP, inventory control and data access control.

Fuzzy extractors were developed for radio-based signals to achieve high consistency. Euclidean metric fuzzy extractor and Hamming metric fuzzy extractor were designed for different location measurement errors. Adequate quantization step should be chosen as it determines the system performance. FAR and FRR can be traded off by varying the quantization steps of location features. We used Loran-C real data to show that the Euclidean metric fuzzy extractor significantly improves the reproducibility of a generated location tag. In addition we proved that the Loran-C location features can achieve high spatial variation using measurements at three different sites, a parking structure, a soccer field and an office building. In addition, we gave evidence that the tag is unpredictable from a distance, which is beneficial to location-based security applications.

## Figures and Tables

**Figure 10.**
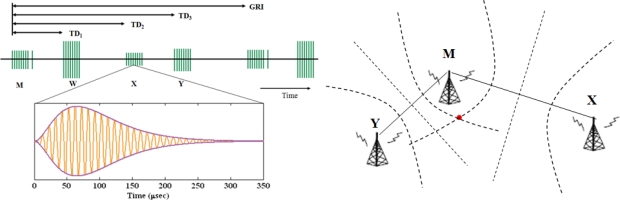
**(a)** Loran chain signals and ideal pulse (left); **(b)** hyperbolic positioning (right).

**Figure 11.**
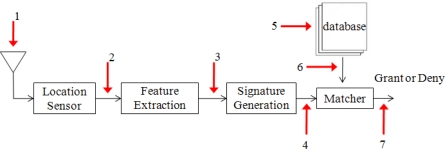
Threat model of generic attacks.

## Appendix A. Loran Background

Loran is a navigation system that operates at 100 kHz. It was developed during World War II and played a significant role in marine navigation [1]. In this section we present the background on Loran and its operations.

### A.1. Basics of Loran

Loran provides position using differences in the arrival time of radio frequency (RF) signals from various stations. Loran stations broadcast pulsed signals in groups called chains, each of which provide coverage to a particular geographic region. Each chain consists of one master station M and several secondary stations, which use letters W, X, Y, and Z. Secondary stations have eight pulses while a master station has nine pulses and the last pulse is used for identification of the master stations. The pulses of each chain are broadcasted repetitively in a constant time interval called group repetition interval (GRI). The repetition interval of each GRI is designed to be different for cross-rate interference rejection. The pulse separation of a station is 1,000 μsec while the last pulse in the master station is 2,000 μsec away from the eighth pulse. The west coast chain is GRI 9940 and its pulses broadcast every 0.0994 seconds. Loran stations are synchronized and the timing of the transmission of stations are controlled by System Area Monitor. Figure 10 illustrates a Loran chain and ideal pulse.

### A.2. Hyperbolic Positioning

Loran uses hyperbolic positioning technique to estimate one’s location. The basic idea is illustrated in Figure 10(b) for 2-D positioning. The red dot indicates a user’s location. The dotted lines represent the line of position (LOP), determined from the received time difference of each station. A minimum of three stations are needed in order to form two equations and solve latitude and longitude, which are the unknowns here. More stations can be used to resolve ambiguity thus provide better geometry and accuracy for the position estimation.

### A.3. Ninth-Pulse Modulation

The current proposal of LDC is ninth-pulse modulation [18]. The modulation is chosen to minimize the impacts on the current operational Loran signal. An additional pulse is inserted after the eighth pulse of pulse group of secondary stations with a minimum of 1,000 μsec separation. Third-two state Pulse Position Modulation (PPM 32) is used to change the time delay of the ninth pulse after the eighth navigation pulse. Each ninth-pulse carries five data bits.

*Loran message structure.* Under the current proposed ninth pulse communications, each Loran message has 120 bits and consists of a 4-bit header, a 41-bit payload, and 75-bit parity component [18]. The Reed-Solomon codes are used for parity check. This forward error correction coding method provides error correction capacity and integrity. It provides to ability to align the message and to verify that the message has been validly decoded with high probability.

*Demodulation.* The performance of demodulation technique in the presence of noise determines the required SNR and signal power necessary to receive data. One demodulation technique to demodulate 9^th^ pulse data is matched filter [18]. A matched filter performs convolutions of a time-reversed version of a reference signal with the input signal. By multiplying the input signal with a time shifted version of the reference signal and integrating the product, the maximum of the integrals is the demodulated symbol.

## Appendix B. Short Survey on Signal Authentication

The main challenge of secure broadcast communication is source authentication, and the problem is complicated by untrusted or uncertified users and unreliable communication environments. Source authentication helps the receivers to verify the received data originates from the source and has been modified in transit.

In this section we have a survey on authenticating radio navigation signals using cryptographic authentication algorithms. We will use the U.S. satellite navigation system Global Positioning System (GPS), the upcoming European satellite navigation system Galieo and Loran as examples to study different schemes and implementations of signal authentication. Even though the history of radio navigation system can go back to 1940s when MIT first developed Loran, the radio navigation signals were not self-authenticated until GPS was operational.

### B.1. GPS Signal Architecture for Military

The system was initially designed for Military use. The GPS signal consists of spreading code or pseudo-random noise (PRN) code for ranging, a RF carrier and navigation data. The military signal has different architectures from the civil signal: faster chipping rate and longer period on the spreading code for higher position precision; encrypted codes for protection against spoofing threats. The precision code is called P-code and together with the encrypted version Y-code, called P(Y) codes. Only military authorized users have the access of the Y-code [19]. We know nothing about the encrypted version ranging code.

### B.2. Proposed Authenticated GPS Signal for Civil Navigation

The current civil signal has neither encrypted nor authenticated mechanism. Logan Scott proposed three levels of authentication on civil navigation system to detect spoofed signals and make it hard for a spoofer to generate valid signals [20].

*Data message authentication.* The first method is to authenticate navigation data messages using public key digital signature. The private key is only available to the control segment and space segment and used to sign the data messages. The public key is available to every GPS user. Spoofers can only authenticate but not generate false messages. Currently there are six message types for positioning and error correcting. A new “Type 7 authentication message” is proposed to authenticate the GPS data messages. This message would be broadcasted once every 5 minutes. Users won’t be able to authenticate until receive both data and authentication messages.

*Public spread code authentication.* However, one navigation bit is twenty times longer than one PRN code, and this short PRN makes this authentication scheme problematic. Spoofers can simply delay the PRN codes while the authenticated navigation messages are still valid and victim receivers would not be able to detect this small delay in time. Level 2 authentication is to interleave a spread spectrum security codes (SSSC) with normal spreading codes. The digital signatures would be broadcasted in the navigation messages. The receiver would not be able to track until the digital signature is received, generate a reference SSSC, and despread the received SSSC to validate the signal. The received GPS signal has extremely low power so dispreading is a very powerful technique to detect the signal. Technically, it is impossible for spoofers to know the actual SSSC without despeading it because the code is buries below the thermal noise.

*Private spreading code authentication.* The third scheme is similar to level 2 authentication. A tamper resistant Civil Antispoof Security Module is used to process private SSSC. This private SSSC will be transmitted with respect to the authentication messages that carry the digital signature; thus the authentication time is short

### B.3. Proposed Authenticated Galileo Signal

Galileo is a European satellite navigation system under civilian control. Several test satellites have been launched and the system is expected to be fully operational by 2013 [21]. The authentication scheme and its implementation have not been finalized yet. The candidate schemes are from a number of existing and proposed authentication methods described above. Galileo will provide different services: Open Service (OS), Safety of Life Service (SoL), Commercial Service (CS) and Public Regulated Service (PRS). OS will have neither authentication nor encryption; SoL will provide authenticated navigation data; CS will encrypt the navigation data; PRS will use both encrypted ranging codes and navigation messages. This results in that the services are different in the levels of integrity and security [22].

### B.4. TESLA on eLoran

Unlike GPS and Galileo, Loran does not have spreading codes but measures delays of pulses for ranging so the SSSC scheme cannot be applied on Loran. The enhanced or modernized Loran has a data channel that carries Loran messages. Therefore, we propose TESLA to authenticate these data messages to insure the integrity of the data and authenticity of the source. One important characteristic of TESLA is one-way key chains: One chain for MAC key generation and the other chain for transmission. The key chain is broadcasted in the reverse order of the construction. To ensure security, the last computed key or first transmitted key should be either embedded or delivered to the receiver via a secure channel. This key will be updated periodically as the key chain has finite length but the broadcast of navigation data is continuous in time. The update period depends on the key chain length and other practical implementation concerns.

## Appendix C. Generic Attacks

This section discusses why the tamper-resistant device and self-authenticated signals are important for the protocol. Without tamper resistance, attack can break the system in many different ways. A threat model of generic attacks is illustrated in Figure 11. The diagram consists of analog segment, digital segment and binary segment. The analog segment includes the RF antenna and location sensor that capture, condition and digitize the RF signals. The digital segment extracts the location features and computes a location tag.

The binary segment has the database and matcher, and performs the matching process to verify the authenticity of the computed location tag. The red arrows with labels represent the generic cryptographic attacks discussed as follows:

1. *Insecure RF signals.* RF signals are vulnerable to jamming and spoofing, which may lead to weaknesses in the designed location-based security systems. The purpose of a location tag is to provide more security to a security system. As such, it is important that every linkage of the location-based security chain is secure. This includes not only the protocol itself but also the broadcast of RF signal. Jammers are radio transmitters that electromagnetically overwhelm the radio navigation signals. On the other hand, spoofers trick the navigation receiver into reporting a location other than the true location of the receiver. It is difficult to jam or spoof Loran-C signals over the air in the far field [23] because such an attack would require significant power and infrastructure. Hence it is either difficult or reasonably detectable. Near field spoofing is a different threat but it requires being reasonably close and potentially exposes the attacker. When an attacker is close enough to the legitimate user, he can simulate RF signals to forge the correct location tag. The security of the RF navigation signal can be provided by message authentication, for example, TESLA as we proposed on eLoran. One goal is to prevent the user from being fooled into believing that a message comes from a particular source when this is not the case. Another goal is to allow the receivers to verify whether the messages have been modified during transmission.
2. *Replay attack.* Attackers may use real authenticated Loran signals to bypass the signal source verification but modify the received Loran signals and replay to spoof the authentication device. The signals after the location sensor is digital. This attack requires the attackers to have signal process skills to modify the location features carried on the RF signals. For instance, attackers can re-position RF pulses to modify the time-of-arrival and other parameters of the incoming signals.
3. *Tamper with features.* The location feature values can be replaced by the ones selected by attackers. This still requires the attackers to bypass the source verification of the authenticated signals.
4. *Brute force attack.* Attackers generate all the possible combinations of the binary tags and replace the tag computed from the received location information with one selected. To protect against this attack, we need location information has high entropy to result in long location tag. The Loran location information measure will be discussed in the later section.
5. *Tamper with location tag database.* The attacks on the template database include adding a new template, modifying an existing template, removing templates, copying template data for secondary uses, *etc.*
6. *Man in the middle attack.* The transmission medium between the template database and the matcher is similarly vulnerable. This can result in an alteration of the transmitted template.
7. *Override the final decision.* Results from the matcher (accept or reject) can be overridden by attackers. If this is a DoS attack, attackers can change the final decision by producing a sufficiently large number of errors to the system. The spoofing attacks type 2 through 7 can be defeated by using a tamper-resistant device.
